# New morphological and molecular data for *Xystretrum solidum* (Gorgoderidae, Gorgoderinae) from *Sphoeroides testudineus* (Tetraodontiformes, Tetraodontidae) in Mexican waters

**DOI:** 10.3897/zookeys.925.49503

**Published:** 2020-04-08

**Authors:** Andrés Martínez-Aquino, Jhonny Geovanny García-Teh, Fadia Sara Ceccarelli, Rogelio Aguilar-Aguilar, Víctor Manuel Vidal-Martínez, M. Leopoldina Aguirre-Macedo

**Affiliations:** 1 Facultad de Ciencias, Universidad Autónoma de Baja California, Carretera Transpeninsular 3917, Fraccionamiento Playitas, Ensenada, Baja California 22860, México Universidad Autónoma de Baja California Ensenada Mexico; 2 Laboratorio de Patología Acuática, Departamento de Recursos del Mar, Centro de Investigación y de Estudios Avanzados del Instituto Politécnico Nacional, Unidad Mérida, Cordemex, Carretera Antigua a Progreso Km. 6, Mérida, Yucatán, 97310, México Centro de Investigación y de Estudios Avanzados del Instituto Politécnico Nacional Merida Mexico; 3 Departamento de Biología de la Conservación, CONACYT-Centro de Investigación Científica y de Educación Superior de Ensenada, Carretera Ensenada-Tijuana, Ensenada, Baja California, 22860, México CONACYT-Centro de Investigación Científica y de Educación Superior de Ensenada Ensenada Mexico; 4 Departamento de Biología Comparada, Facultad de Ciencias, Universidad Nacional Autónoma de México, Circuito Exterior s/n, Ciudad Universitaria, 04510, Ciudad de México, México Universidad Nacional Autónoma de México Ciudad de Mexico Mexico

**Keywords:** COI, molecular phylogenetics and systematics, parasites of marine fishes, scanning electron micrographs, 28S

## Abstract

Adults of trematodes in the genus *Xystretrum* Linton, 1910 (Gorgoderidae, Gorgoderinae) are parasites found exclusively in the urinary bladders of tetraodontiform fishes. However, limited and unclear morphological data were used to describe the type species, *X. solidum* Linton, 1910. Here, we present the first detailed morphological information for a member of *Xystretrum*. Morphological characters were described using light and scanning electron microscopy (SEM) of *Xystretrum* specimens from *Sphoeroides testudineus* (Linnaeus) (Tetraodontiformes, Tetraodontidae), collected at six localities off the northern Yucatan Peninsula coast of the Gulf of Mexico. We also compared sequence fragments of the 28S (region D1–D3) ribosomal DNA and mitochondrial Cytochrome c oxidase subunit 1 (COI) gene with those available for other gorgoderine taxa. We assigned these *Xystretrum* specimens to *X. solidum*, despite the incompleteness of published descriptions. The data provide a foundation for future work to validate the identities of *X. solidum*, *X. papillosum* Linton, 1910 and *X. pulchrum* (Travassos, 1920) with new collections from the type localities and hosts. Comparisons of 28S and COI regions described here also provide an opportunity to evaluate the monophyletic status of *Xystretrum*.

## Introduction

Linton (1910) proposed the genus *Xystretrum* Linton, 1910 (Gorgoderidae, Gorgoderinae) to include two new trematode species, *X. solidum* Linton, 1910 (type species) and *X. papillosum* Linton, 1910, which, as adults, are parasites of tetraodontiform fishes of the families Balistidae (*Balistes capriscus* Gmelin [as *B. carolinensis*] from off Bermuda) and Ostraciidae (*Lactophrys triqueter* (Linnaeus) from Dry Tortugas, Florida, USA). Unfortunately, the original descriptions of both species are incomplete and unclear. This has resulted in taxonomic confusion when new species have been proposed. Several *Xystretrum* species have subsequently been reported, synonymized and later resurrected by some (but not all) authors, while the validity of others remain doubtful (Linton 1907; MacCallum 1917; Manter 1947; Yamaguti 1971; Siddiqi and Cable 1960; Nahhas and Cable 1964; Overstreet 1969).

The most reliable list of species of *Xystretrum* available is the public resource database of the World Register of Marine Species (WoRMS 2020), which lists 14 accepted species. Of these species, *X. solidum* and *X. papillosum*, along with *X. pulchrum* (Travassos, 1920) Manter 1947, are reported from the Northwest Atlantic Ocean and Gulf of Mexico (Linton 1910; Travassos 1922; Manter 1947; Mendoza 2016). However, *X. pulchrum* was also inadequately described from *Sphoeroides testudineus* (Linnaeus) (Tetraodontiformes, Tetraodontidae) collected in the southwestern Atlantic Ocean (Manquinhos State, southeastern Brazil) (Travassos 1922; Manter 1947) and its incomplete description has generated synonyms (e.g., Siddiqi and Cable 1960; Overstreet 1969). *Xystretrum pulchrum* was reported from the type locality of *X. papillosum* (i.e., Tortugas, Florida) and from the North Pacific Ocean (Hawaii), but the morphological data used to separate the two species are questionable (Travassos 1922; Manter 1947; Hanson 1955; Yamaguti 1970). Thus, the morphological descriptions of *X. solidum*, *X. papillosum* and *X. pulchrum* remain incomplete.

Despite the scarce taxonomic information from the western Atlantic, Overstreet et al. (2009) reported *X. solidum* parasitizing the kidneys and urinary bladder of five tetraodontiform fish species from four families (i.e., *B. capriscus* [Balistidae], *L. triqueter* [Ostraciidae], *S. spengleri* Bloch, *S. testudineus* [Tetraodontidae] and *Stephanolepis hispidus* (Linnaeus) [Monacanthidae]) distributed from Bermuda, the Caribbean Sea and the North Gulf of Mexico to the Atlantic coast of South America.

Cutmore et al. (2013) provided the first genetic sequence data for an Atlantic species, tentatively identified as *X. solidum*, from *S. testudineus* collected in the Florida Keys near (200 km in an approximately straight line) Dry Tortugas, the type locality of *X. papillosum*. Recently, Pérez-Ponce de León and Hernández-Mena (2019) provided a second genetic sequence published as *X. solidum* from *Balistes vetula* (Linnaeus) (Balistidae) from Puerto Morelos, Quintana Roo, Mexico, from the Mexican Caribbean.

Several *S. testudineus* from the coasts and lagoons of the Northern Yucatan Peninsula, Mexico were examined for parasites between 1995 and 2013. Gorgoderids were recovered from the urinary bladder of these hosts and preliminarily identified as *Phyllodistomum* sp. (Tello 1999; Pech et al. 2009; Sosa-Medina et al. 2014, 2015). Following a study program to fully characterize parasite biodiversity (Pérez-Ponce de León and Nadler 2010; Nadler and Pérez-Ponce de León 2011), DNA sequences of the 28S gene were obtained from these “*Phyllodistomum* sp.” and compared to GenBank sequences available for gorgoderines. A high similarity (BLAST scores) between the “*Phyllodistomum* sp.” and trematodes tentatively identified as *X. solidum* by Cutmore et al. (2013) suggests that records for these *Phyllodistomum* should be reassigned to the genus *Xystretrum*.

As Nadler and Pérez-Ponce de León (2011) pointed out, phylogenetic analyses are essential for correctly characterizing a species (including cryptic species) and data from morphological approaches; e.g., morphology, morphometry and microphotographs of scanning electron microscopy (SEM) should be corroborated using molecular-based results. Both morphological and molecular information are lacking for three species of *Xystretrum* found in the Atlantic Ocean (i.e., *X. solidum*, *X. papillosum* and *X. pulchrum*). The intention here is to provide morphological descriptions to support the reassignment of trematodes previously identified as *Phyllodistomum* to *Xystretrum* and to provide new morphological and sequence data to facilitate future revisions of the genus *Xystretrum*.

## Materials and methods

### Collection of hosts and trematodes

**Figure 1:**
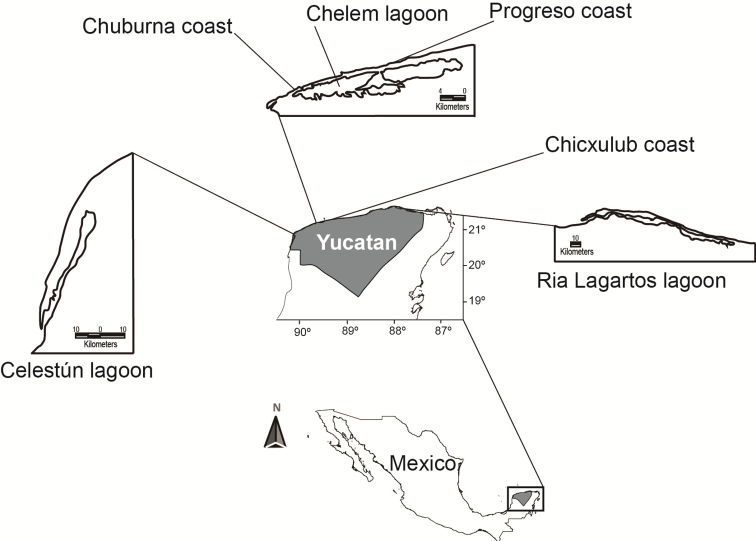
. Northern Yucatan Peninsula, Mexico, showing localities where the specimens of *Xystretrum solidum* were collected.

Trematode specimens in this study were collected from the urinary bladder of *Sphoeroides testudineus*. Hosts were collected between 1998 and 2016 (collection permit PPF/DGOPA-070/16 issued by Comisión Nacional de Acuacultura y Pesca, Mexico) at six localities in and off the northern Yucatan Peninsula, Mexico: Celestún tropical lagoon (20°45'N, 90°22'W, June 2005, August 2012, January 2016), Chelem lagoon (21°15'N, 89°45'W, August 2005, March 2007), Ría Lagartos lagoon (21°22'N, 87°30'W, July 2005), Chuburna port (coastal area) (21°15'N, 89°48'W, March 2005), Progreso port (coastal area) (21°16'N, 89°39'W, August 2006), and Chicxulub port (coastal area) (21°17'N, 89°36'W, June 2009) (Fig. 1). Specimens were fixed in 4% hot formalin for morphological treatment or scanning electron micrographs (SEM), or in absolute ethanol for molecular analyses.

### Morphological data and morphometric analyses

Unflattened trematode specimens were stained with Mayer’s paracarmine and mounted on permanent slides using Canada balsam. Specimens were measured, and drawings were made with the aid of a drawing tube attached to an Olympus BX50 microscope; measurements are presented in micrometres (μm) as ranges followed by the means in parentheses. For the SEM study, specimens were dehydrated through a graded series of ethyl alcohols and critical point dried with carbon dioxide. Specimens were mounted on metal stubs with silver paste, then coated with gold and examined in a Philips XL30 ESEM for variable pressure SEM at 10 kV. Trematodes were identified following/contrasting the taxonomic criteria of Linton (1907; 1910), Travassos (1922), Manter (1947; 1972), Winter (1959), Overstreet (1969), Yamaguti (1971), Campbell (2008) and Madhavi and Bray (2018). Holotype, labelled as *X. papillosum* (No. 1321174; now in the Smithsonian Institution National Museum of Natural History (NMNH) (ex USNM Helm. Coll. No. 8426) from *Lactophrys triqueter* from Dry Tortugas, Florida, USA, was studied to compare with the newly collected specimens. The *X. solidum* holotype described by Linton in 1907 was not found in the scientific collections in the USA, Europe or Australia, and should be considered lost. Several specimens collected for morphological analysis were deposited as voucher specimens in the Colección Helmintológica del CINVESTAV (CHCM), Departamento de Recursos del Mar, Centro de Investigación y de Estudios Avanzados del Instituto Politécnico Nacional, Unidad Mérida, Yucatán, Mexico (Tab. 1). Morphological measurements obtained in this study were compared with those of the 14 congeneric *Xystretrum* spp. (Suppl. material 1: Tab. S1).

**Table 1.** Localities sampled (from east to west) for *Sphoeroides testudineus*, the host species of *Xystretrum solidum*, from the Yucatan Peninsula, Yucatan, Mexico. LM = Total number of measured individuals of *X. solidum* used for morphometric studies on light microscope slides. SEM = Total number of individuals of *X. solidum* used for scanning electron micrograph studies. CHCM = Voucher number from the Colección Helmintológica del CINVESTAV (CHCM) for specimens studied in this work.

**Localities**	**LM**	**SEM**	**CHCM**
Celestún	4	1	529
Chuburna	3	4	530
Chelem	1	1	531
Progreso	6	2	532
Chicxulub	1	–	533
Ría Lagartos	2	3	534

### DNA extraction, PCR amplification and sequencing

Deoxyribonucleic acid (DNA) was extracted from individual adult trematodes; DNA extraction was performed using the DNeasy blood and tissue extraction kit (Qiagen, Valencia, CA, USA) following the manufacturer’s instructions. Partial sequences of the 28S (region D1–D3) ribosomal DNA were amplified by Polymerase Chain Reaction (PCR) (Saiki et al. 1988) using 28sl fwd (5´-AAC AGT GCG TGA AAC CGC TC- 3´) (Palumbi 1996) and LO rev (5´-GCT ATC CTG AG(AG) GAA ACT TCG- 3´) (Tkach et al. 2000). The primers JB3 fwd (5´-TTT TTT GGG CAT CCT GAG GTT TAT- 3´) (Morgan and Blair 1998) and CO1R trema rev (5´ -CAA CAA ATC ATG ATG CAA AAG G- 3´) (Miura et al. 2005) were used for the COI fragment. The reactions were prepared using the Green GoTaq Master Mix (Promega). This procedure was carried out using an Axygen Maxygen thermocycler. The PCR cycling conditions were as follows: for COI, an initial denaturing step of 3 min at 94 °C, followed by 35 cycles of 92 °C for 30 sec, 47 °C for 45 sec and 72 °C for 90 sec, and a final extension step at 72 °C for 10 min; for 28S, an initial denaturing step of 5 min at 94 °C, followed by 35 cycles of 92 °C for 30 sec, 50 °C for 45 sec and 72 °C for 90 sec, and a final extension step at 72 °C for 10 min. The PCR products were analyzed by electrophoresis in 1% agarose gel using the TAE 1X buffer and observed under UV light using the QIAxcel Advanced System. The purification and sequencing of the PCR products were carried out by Genewiz, South Plainfield, NJ, USA (https://www.genewiz.com/).

### Molecular data and phylogenetic reconstruction

**Figure 2.**
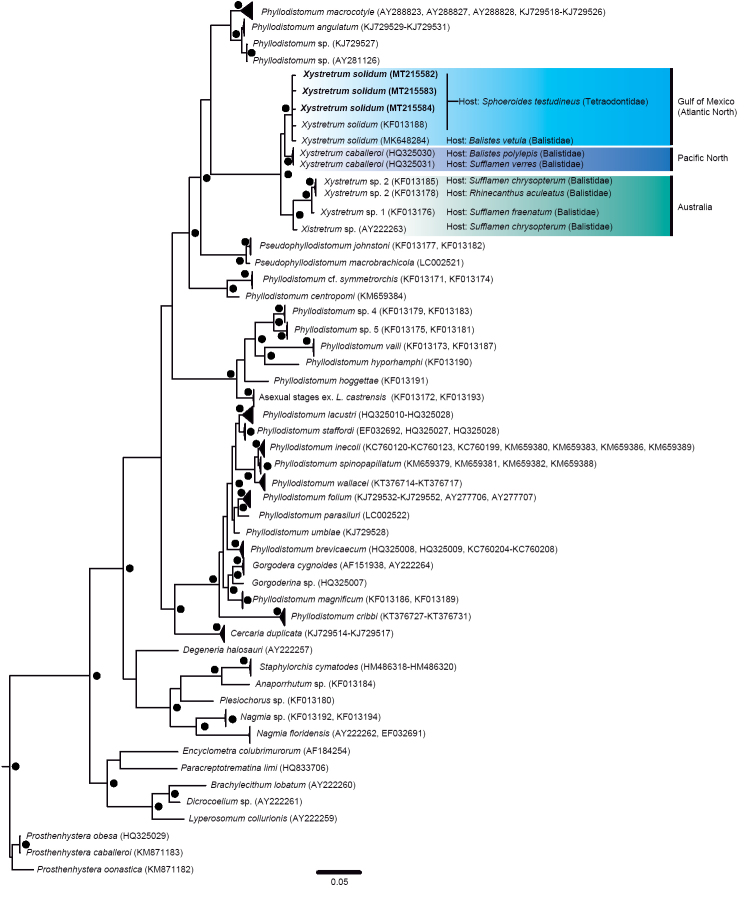
Phylogenetic tree obtained using Bayesian inference for the 28S rRNA dataset. The scale bar represents the number of nucleotide substitutions per site. GenBank accession numbers of the new sequences of *Xystretrum solidum* are shown in bold. Filled circles above/below branches and at the nodes represent Bayesian Posterior Probability ≥ 0.95.

**Figure 3.**
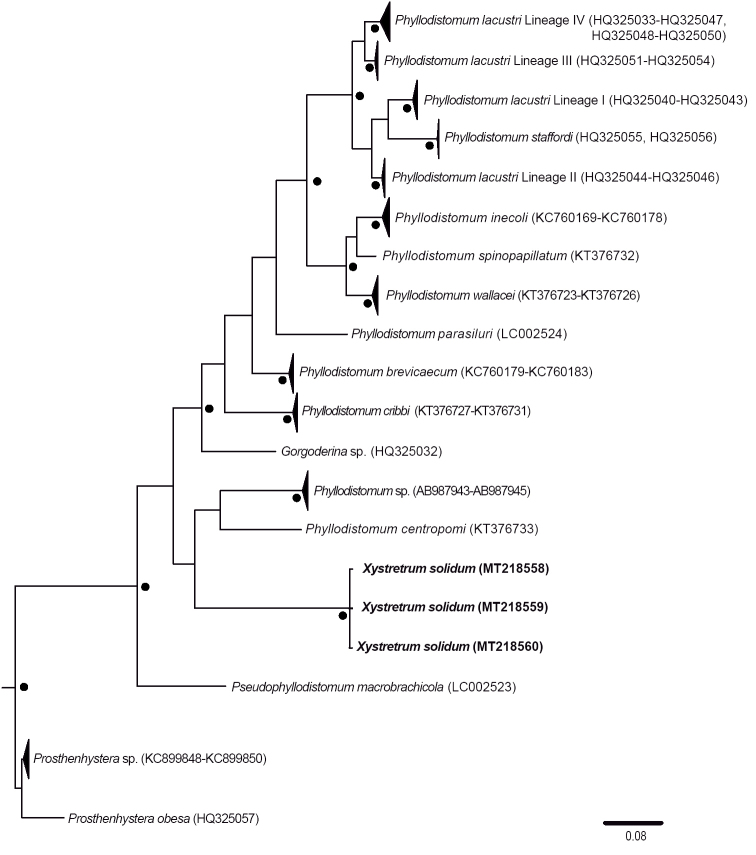
Phylogenetic tree obtained using Bayesian inference for the COI dataset. The scale bar represents the number of nucleotide substitutions per site. GenBank accession numbers of the new sequences of *Xystretrum solidum* are shown in bold. Filled circles above/below branches and at the nodes represent Bayesian Posterior Probability ≥ 0.95.

To obtain the consensus sequences of specimens of *Xystretrum*, we assembled and edited the chromatograms of forward and reverse sequences using the Geneious Pro v.5.1.7 platform (Kearse et al. 2012). The 28S and COI partial sequences generated during this study were aligned with sequences of gorgoderids and representative outgroup sequences of members of the Allocreadiidae, Callodistomidae, Dicrocoeliidae and Encyclometridae (see GenBank accession numbers in Figs 2, 3) used previously by Cutmore et al. (2013), Martínez-Aquino et al. (2013), Petkevičiūtė et al. (2015) and Urabe et al. (2015), using an interface available in MAFFT v.7.263 (Katoh and Standley 2016), an “auto” strategy and a gap-opening penalty of 1.53 with Geneious Pro, and a final edition by eye in the same platform. The Gblocks Website v.0.91b (Castresana 2000; Talavera and Castresana 2007) was used to remove ambiguously aligned regions of 28S. To evaluate the sequence and molecular marker utility for phylogenetic analyses at the intended taxonomic level (family level for the complete-outgroup dataset and genus level for the *Xystretrum* dataset), we tested the nucleotide composition homogeneity within each data alignment (28S and COI matrix data), using chi-squared metric provided in the program TreePuzzle v.5.3.rc (Schmidt et al. 2002). The software jModelTest v.2.1.3 (Darriba et al. 2012) was used to select evolution models through the Bayesian Information Criterion (BIC) (Schwarz 1978) for each dataset separately (28S and COI). The nucleotide substitution model that best fit the 28S dataset was TVM+I+G (Posada 2003). The COI dataset was partitioned into first-, second- and third-codon positions with the appropriate nucleotide substitution model implemented for each codon position (TrN+I for the first [Tamura and Nei 1993]; TPM3uf+I for the second [Kimura 1981]; and HKY+I for the third codon position [Hasegawa et al. 1985]). Furthermore, the net evolutionary distances between *Xystretrum* taxa, using *p*-value with variance estimation, with the Bootstrap method (500 replicates) and with a nucleotide substitution (transitions + transversions) uniform rate, were estimated for the 28S fragment in MEGA v.7.0 (Kumar et al. 2016).

Phylogenetic trees were reconstructed for each gene separately (28S and COI), to test the monophyly of *X. solidum* analyzed in this study. Phylogenetic tree reconstructions were carried out using Bayesian Inference (BI) in MrBayes v.3.2.3 (Ronquist et al. 2012), with two parallel analyses of Metropolis-Coupled Markov Chain Monte Carlo (MCMCMC) for 20 × 10^6^ generations each. Topologies were sampled every 1000 generations and the average standard deviation of split frequencies was observed to be less than 0.01, as suggested by Ronquist et al. (2012). A majority consensus tree with branch lengths was reconstructed for the two runs after discarding the first 5000 sampled trees. The robustness of the clades was assessed using Bayesian Posterior Probability (PP), where PP > 0.95 was considered strongly supported. The Bayesian phylogenetic reconstructions were run through the CIPRES Science Gateway v.3.3 (Miller et al. 2010).

## Results

**Figure 4.**
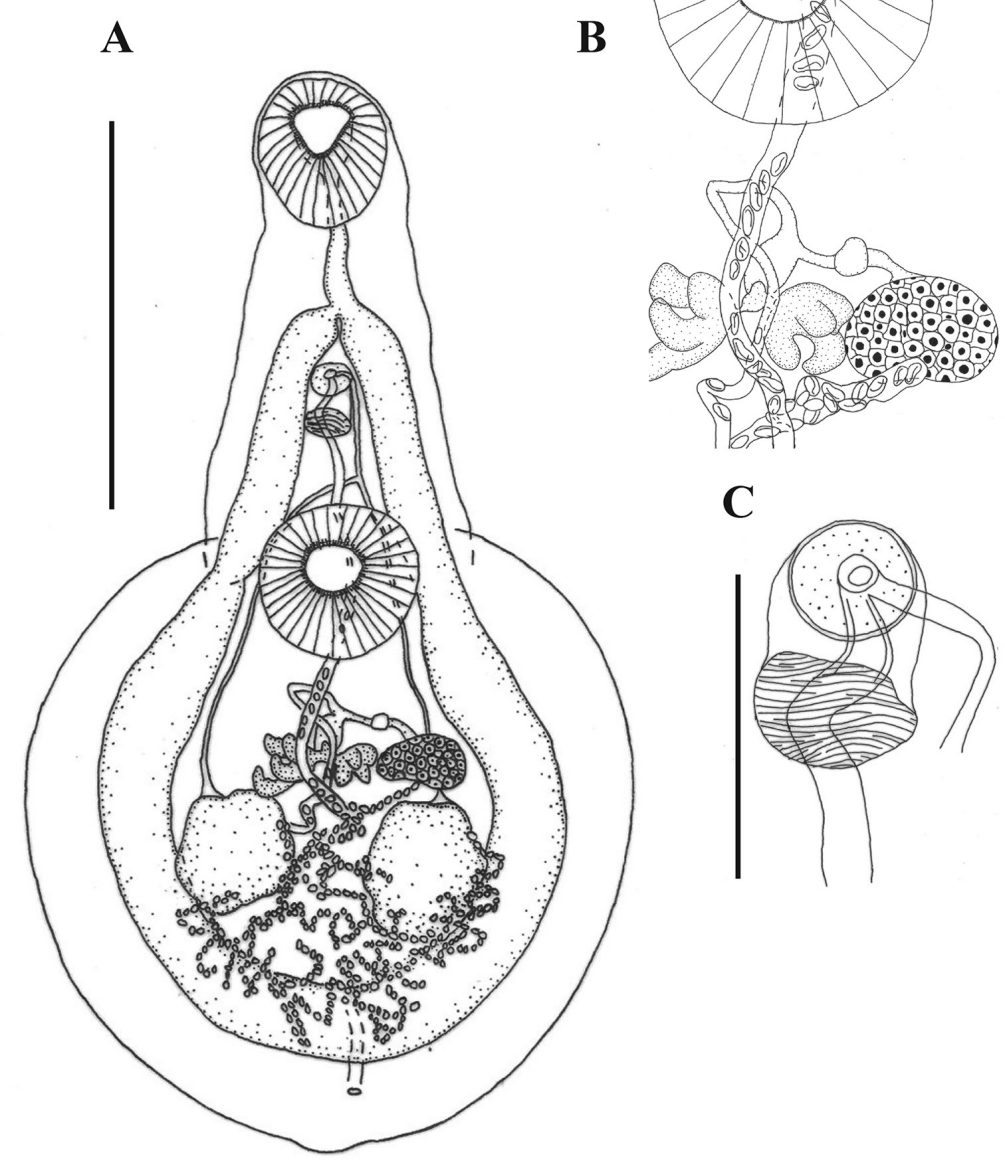
Line drawings of the 532 CHCM-voucher of *Xystretrum solidum* from the urinary bladder of *Sphoeroides testudineus***A** whole specimen (ventral view) **B** details of reproductive organs **C** details of genital atrium. Scale bars: 1000 µm (**A**); 250 µm (**B–D**).

**Figure 5.**
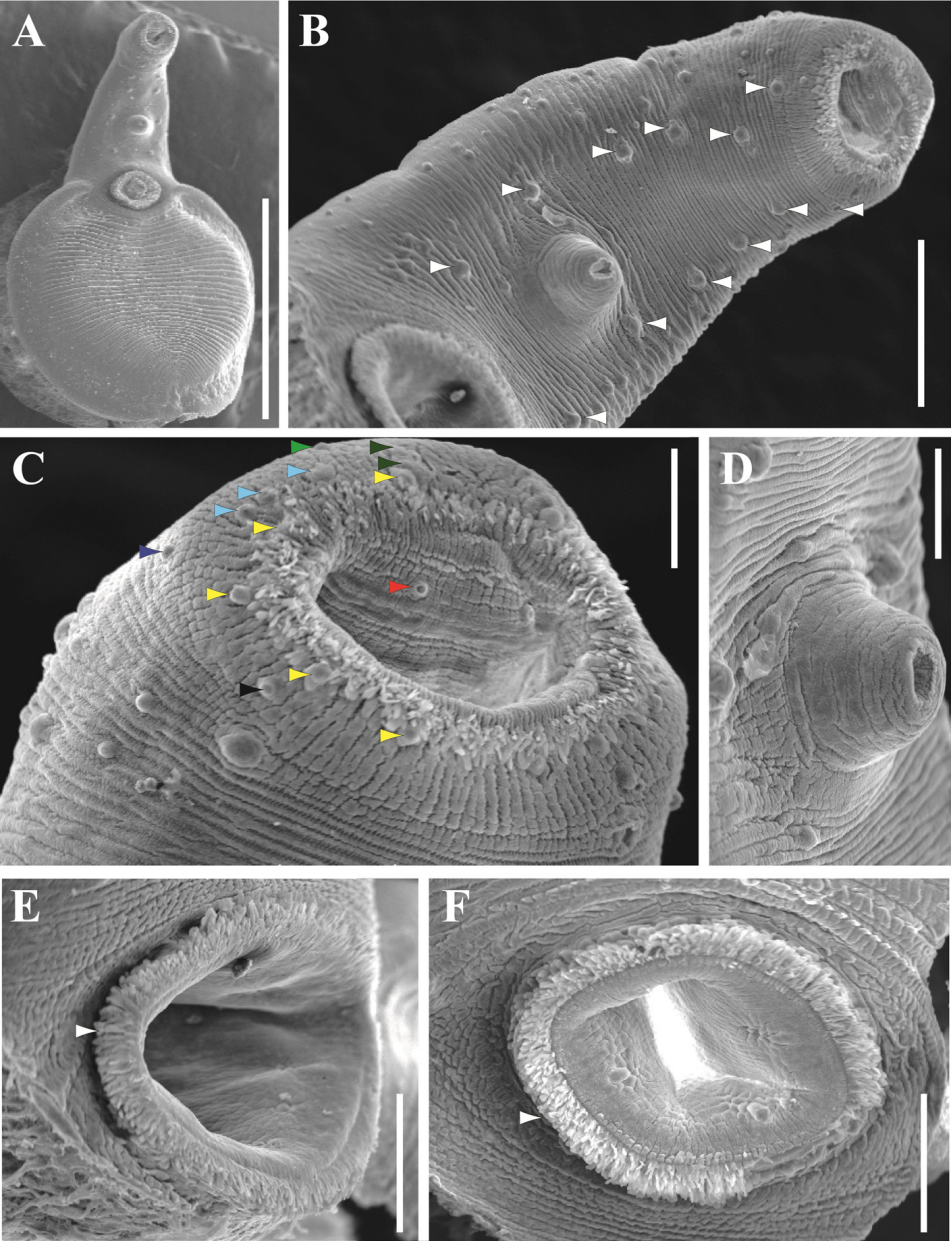
Scanning electron microscopy (SEM) images of *Xystretrum solidum* (from three specimens collected at Progreso Port, Yucatan, Mexico) **A** whole adult specimen (ventral view) with scattered rosette papillae on forebody **B** forebody, showing 6 pairs of robust papillae (white arrowhead) **C** oral sucker, showing 13 pairs of papillae: 5 on interior margin surrounding mouth (yellow arrowhead); one posterolateral to interior margin (dark blue arrowhead); three anterolateral to interior margin (light blue arrowhead); 2 on stylet scar (dark green arrowhead); one lateral to stylet scar (light green arrowhead); one on posterior external margin of oral sucker (black arrowhead); one inside of mouth (red arrowhead) (only right hand side papillae are indicated) **D** genital atrium detail **E** ventral sucker (side view), showing long papillae on inner margin (white arrowhead) **F** ventral sucker (ventral view), showing long papillae on inner margin (white arrowhead). Scale bars: 1000 µm (**A**); 200 µm (**B**); 500 µm (**C, D**); 100 µm (**E, F**). For more details of observed characters by SEM from other localities analyzed in this study, see Suppl. material 2: Figure S1.

Specimens analyzed were assigned to *Xystretrum solidum* (Figs 4, 5 and Suppl. material 2: Fig. S1). Measurements are of 17 individuals from six localities, and details of the body surface are of 11 gravid specimens from five localities (Tab. 1). New taxonomic and morphometric data: Body flask-shaped, with smooth lateral margins in forebody, 1870–3520 (2750) long, 1020–2100 (1550) wide. Forebody long, narrow, sub-cylindrical, 800–1600 (1170) long, 420–900 (730) wide, representing 35–47% (43%) of total body length. Tegument without spines. Forebody tapered anteriorly. Surface of forebody with elongated and rosette-type papillae (Fig. 5A, B and Suppl. material 2: Fig. S1C). Inner margin of oral sucker covered by fringe arrangement of elongated papillae (Fig. 5C, and Suppl. material 2: Fig. S1A, D, J). Subterminal oral sucker rounded, 200–500 (360) long, 230–450 (330) wide, bearing 14 pairs of well-developed rosette-type papillae, arranged in five pairs on interior margin surrounding mouth; one pair on posterolateral on interior margin; three pairs on anterolateral to interior margin; two pairs on stylet scar; one pair lateral to stylet scar; one pair on posterior external margin of oral sucker; and one pair inside mouth (Fig. 5C; Suppl. material 2: Fig. S1A, B, D, E, J). Ventral region between oral and ventral suckers with six pairs of robust papillae, arranged in two columns, plus several pairs of small papillae (between six and 10) at the lateral borders of this region, and six to seven additional pairs distributed heterogeneously (Figs 5B and Suppl. material 2: Fig. S1C). Hindbody oval in outline, foliaceus, corrugated and demarcated by folds, 1000–2050 (1590) long, 1020–2100 (1550) wide; papillae absent in this region, except for long papillae covering inner margin of ventral sucker, but marked grooves are present (Fig. 5E, F and Suppl. material 2: Fig. S1G, H). Ventral sucker muscular, slightly pre-equatorial, 270–560 (430) long, 300–580 (450) wide. Sucker ratio 1:1.30 (1.05–1.89). Pharynx absent. Oesophagus 150–260 (200) long, 50–130 (90) wide. Intestinal bifurcation in first third of body, 360–690 (530) from anterior end. Caeca long, narrow, running laterally into hindbody, joining close to posterior extremity of body forming cyclocoel, 3190–6130 (4580) long, 90–210 (160) wide; post-caecal space 120–330 (200) long. Testes two, irregular, slightly symmetrical, rounded, inter-caecal in middle of hindbody; left testis 210–420 (300) long, 140–460 (310) wide; right testis 210–460 (300) long, 240–480 (330) wide. Efferent ducts anterior to ventral sucker, forming *vas deferens*. Seminal vesicle tubular, posterior to intestinal bifurcation, 120–360 (220) long, 80–130 (100) wide. Pseudosinus-sac present. Genital pore immediately posterior to intestinal bifurcation, 450–980 (690) from anterior extremity. Ovary smooth, oval, sinistral, anterior to right testis, 110–190 (150) long, 140–300 (200) wide. Oviduct connected to common vitelline duct. Vitellarium in two symmetrical, lobed masses (3–4 lobes), conspicuous vitelloduct intersections present in terminal part of second third of the body; masses 140–280 (180) long, 80–290 (160) wide. Uterus distributed in inter-caecal area, forming several loops, sometimes overlapping caeca slightly at level of middle and posterior part of body (Fig. 4A). Eggs elliptical, 30–60 (50) long, 20–30 (20) wide. Excretory vesicle I-shaped; excretory pore subterminal, dorsal, 90–250 (160) from posterior end of body.

Host: *Sphoeroides testudineus* (Tetraodontidae).

Site: Urinary bladder.

Localities: Dry Tortugas, Florida, USA (Gulf of Mexico). New localities from the Northern Yucatan Peninsula, Mexico: Celestún tropical lagoon (20°45'N, 90°22'W), Chelem lagoon (21°15'N, 89°45'W), Ría Lagartos lagoon (21°22'N, 87°30'W), Chuburna port (coastal area) (21°15'N, 89°48'W), Progreso port (coastal area) (21°16'N, 89°39'W), Chicxulub port (coastal area) (21°17'N; 89°36'W).

GenBank accession numbers: 28S rDNA sequences: MT215582–MT215584; COI mtDNA sequences: MT218558–MT218560.

### DNA sequences and dataset analyses

In total, 12 bi-directional 28S and COI sequences were obtained from three individual adults of *X. solidum*. The final lengths (in number of base-pairs) of the 28S ribosomal sequence fragment were 892 (for two sequences) and 899 (for one sequence), with zero genetic variation, either among the new sequences or in the published 28S sequences of *X. solidum* (GenBank accession numbers MK648284 and KF013188). The total alignment length following the Gblocks exclusion was 814 bp. Nucleotide sequence variation in the 28S alignment from gorgoderids (excluding the outgroup taxon) included 330 conserved sites, 483 variable sites, 410 parsimony-informative sites and 73 singleton sites. The COI dataset consisted of 309 bp with a genetic distance of 0.3% between the three mitochondrial sequences. Nucleotide sequence variation (excluding the outgroup taxa) for each partition from COI (first, second and third codon positions) was 70/92/12 conserved, 33/11/91 variable, 29/7/79 parsimony-informative and 4/4/12 single sites, respectively.

### Phylogenetic reconstructions

We inferred the phylogenetic relationships from the 28S and COI sequence matrices separately. The 28S gene dataset contained 46 taxa (150 sequences) and the COI contained 18 taxa (63 sequences). Figures 2 and 3 show the phylogenetic topologies resulting from 28S and COI dataset analyses, respectively. The 28S tree shows that the sequences generated in this study form a clade with the sequences from the material tentatively identified as *X. solidum* (sequence KF013188) by Cutmore et al. (2013) and *X. solidum* (sequence MK648284) from *B. vetula* in the Gulf of Mexico (see below). Furthermore, in the 28S tree, species of *Xystretrum* form a monophyletic group, with high nodal support values (PP ≥ 0.95) (Fig. 2). The COI tree shows that all sequences of *X. solidum* form a clade (Fig. 3). Based on the phylogenetic trees constructed from the 28S dataset, the taxa most closely related to *Xystretrum* spp. are members of the genus *Phyllodistomum* (i.e., *Phyllodistomum angulatum* Linstow, 1907, *P. macrocotyle* Lühe, 1909 and *Phyllodistomum* sp.), whereas the relatives of the species *X. solidum*, based on the COI dataset, were *Phyllodistomum centropomi* Mendoza-Garfias & Pérez-Ponce de León, 2005 and *Phyllodistomum* sp. (Fig. 3). The differences in topology between the two trees are most likely due to the differences in the taxa included in the two datasets. The genetic distance values from the 28S dataset of *X. solidum*, when compared with *Xystretrum* spp., were 2.74 %, 3.50 %, 5.02 % and 5.02 % for *X. caballeroi*, *Xystretrum* sp., *Xystretrum* sp. 1 and *Xystretrum* sp. 2, respectively.

## Discussion

The morphologies of the trematodes examined in this study are consistent with those of the genus *Xystretrum* provided by Campbell (2008); i.e., intestinal caeca forming a cyclocoel, presence of a pseudosinus-sac, and a corrugated hindbody demarcated by folds. This study adds detail to those descriptions by providing new morphological and morphometric data and revealing characters not previously described, such as the number of papillae on the tegument and oral sucker. However, the published descriptions for the species of *Xystretrum* are very basic, particularly from the American Atlantic, i.e., *X. solidum*, *X. papillosum* and *X. pulchrum*. Body size range (i.e., length) is the primary character used to distinguish these three species. *Xystretrum solidum* is the smallest (i.e., 1750), *X. papillosum* is intermediate (i.e., 2100) (a size that corresponds to the samples analyzed in this study) and *X. pulchrum* is the largest (i.e., 4500). However, since there are no data on intraspecific morphological variation for the three species, it is impossible to decide whether body size is sufficient for the correct identification of our specimens. There are several impediments to species-level identification, including: 1) scarce morphological data from congeners, and particularly the limited measurements for *X. solidum* and *X. papillosum*, 2) voucher material (holotype) apparently lost for *X. solidum*, and 3) incongruences in the host specificity patterns previously reported for *Xystretrum* spp. at family level. For these reasons and based on the genetic similarities and the phylogenetic relationships obtained in this study, we agree with the proposal of Cutmore et al. (2013) and identify our samples as *X. solidum*.

Based on the observation of material from this study, plus the holotype of *X. papillosum* (voucher 1321174), we found that *X. solidum* presents a fluted tegument on the hindbody and that along the dorsoventral margin there are short dense fringe papillae (only readily visible using SEM, see Suppl. material 2: Fig. S1I), which were referred to as “hair-like spines” by Manter (1947; page 330) but without mentioning their exact location. The material examined in this study shows a relatively broad range of polymorphism.

It is necessary to collect new *X. solidum* specimens from the original host (i.e., *B. capriscus*) and the type locality (i.e., off Bermuda) to compare their morphological measurements with our samples. Also, it is necessary to explore the possible presence of *X. papillosum* from *L. triqueter* co-distributed with *B. capriscus* off Bermuda, as part of a taxonomic revision of the genus *Xystretrum*, taking into consideration the morphological data presented here. In parallel, future revisions should seek to distinguish or synonymize *X. solidum*, *X. papillosum* and *X. pulchrum*, while being sensitive to the potential presence of cryptic species.

To date, 14 species of the genus *Xystretrum* are considered valid (WoRMS 2020). From a purely biogeographical standpoint, most of these species are non-conspecific with our material, as they have been reported from unique marine regions other than the Gulf of Mexico. This gives them a set of host-associations and biogeographical differences with respect to the remaining species. Thus, species such as *X. chauhani* Ahmad, 1982, *X. manteri* Ahmad, 1982, *X. overstreeti* Ahmad, 1982, *X. srivastavai* Ahmad, 1982 and *X. thapari* Ahmad, 1982 appear to be confined to the Arabian Sea; *X. abalistis* Parukhin, 1964 occurs in the Gulf of Tonkin (South China Sea); *X. triacanthi* Ahmad & Gupta, 1985 occurs off the Indian coast of the Bay of Bengal (Indian Ocean) (Madhavi and Bray 2018); *X. moretonense* Manter, 1972 and *X. plicoporatum* Manter, 1972 inhabit Australian waters (Manter 1972); and *X. caballeroi* Bravo-Hollis, 1953 and *X. hawaiiense* Yamaguti, 1970 are distributed in various parts of the Pacific Ocean (Bravo-Hollis 1953; Winter 1959; Parukhin 1964; Yamaguti 1970; Arthur and Te 2006; Mendoza 2016). An additional marine region, extending through the Western Atlantic from Brazil to Bermuda and the Gulf of Mexico, harbors the species *X. solidum*, *X. papillosum* and *X. pulchrum* described from members of the Balistidae, Ostraciidae and Tetraodontidae, respectively. The fact that the latter species are geographically sympatric and were described some time ago, resulting in a confused taxonomic characterization, suggests that the published records may indicate incorrect host assignments and host localities.

In the phylogenetic tree obtained from the 28S dataset, all members of the genus *Xystretrum* included in the analysis formed a well-supported clade, but without nodal support with their sister clade. A similar result has been reported in previous phylogenetic analyses carried out for similar taxa using the same gene (e.g., Cutmore et al. 2013; Razo-Mendivil et al. 2013; Pérez-Ponce de León et al. 2015; Petkevičiūtė et al. 2015; Urabe et al. 2015; Stunžėnas et al. 2017). Cutmore et al. (2013) detected that the species of *Xystretrum* genus are not closely related to marine representatives of the family Gorgoderidae. At the present time, species of *Xystretrum* appear related to the freshwater *Phyllodistomum* spp. Even though the *Xystretrum* clade did not exhibit a high nodal support based on the 28S dataset in this study, a phylogenetic relationship with freshwater phyllodistomid trematodes was observed, e.g., *Phyllodistomum* sp., *P. angulatum* and *P. macrocotyle* (see also Stunžėnas et al. 2004; Petkevičiūtė et al. 2015). Because of the incomplete dataset of gene sequences for *Xystretrum* spp., the phylogenetic relationships of this genus remain unclear. Based on the 28S phylogenetic tree topology, *X. solidum* (found in Tetraodontidae and Balistidae) seems to be related to *X. caballeroi* (although lacking nodal support). Current phylogenetic information confirms another sub-clade, which includes samples of *Xystretrum* associated with the Balistidae from the Coral Sea, Australia and the Indian Ocean off Western Australia (as *Xystretrum* sp., *Xystretrum* sp. 1 and *Xystretrum* sp. 2 in Cutmore et al. [2013]).

The COI phylogenetic topology shows a clade with *X. solidum* from this study, and the clade formed by the freshwater taxa *P. centropomi* + *Phyllodistomum* sp. (from Urabe et al. [2015]) as sister, although this relationship does not have nodal support. However, based on COI phylogenetic topology plus 28S, it is possible to suggest a diversification of the most recent common ancestor of *Xystretrum* from freshwater to marine environments, and a subsequent diversification in tetraodontiforms via host-switching events. A similar evolutionary process of transition from freshwater to marine environments has also been suggested for other platyhelminth groups (e.g., Álvarez-Presas et al. 2008; Van Steenkiste et al. 2013; Martínez-Aquino et al. 2017).

As indicated by Cutmore et al. (2013), *Xystretrum* occurs only in marine fishes of the order Tetraodontiformes; however, patterns of host specificity at the family level for each species of *Xystretrum* are not currently well-defined. For example, in the reported cases of *X. solidum* from the American Atlantic, Overstreet et al. (2009) recorded *X. solidum* associated with five tetraodontiform fish species included in four families, including the host species from which *X. papillosum* and *X. pulchrum* were described, i.e., *L. triqueter* and *S. testudineus*, respectively. Furthermore, the recently published sequence by Pérez-Ponce de León and Hernández-Mena (2019) (sequences MK648284) suggests that *X. solidum* is associated with both the Tetraodontidae and Balistidae. Although there are efforts to build molecular libraries of parasite biodiversity (Pérez-Ponce de León and Nadler 2010; Nadler and Pérez-Ponce de León 2011), DNA sequences of parasites with little or no morphological support continue to be generated, primarily due to incomplete taxonomic descriptions. Here, we provide molecular sequences supported by detailed morphological description, which can provide a foundation for future comparisons and revisions within *Xystretrum* and the Gorgoderidae.

## Supplementary materials

Supplementary material 1 Table S1 Explanation note Comparative data of relevant taxonomic characters of *Xystretrum solidum* from this study, in contrast to the 14 congeneric *Xystretrum* spp. https://binary.pensoft.net/file/398499This dataset is made available under the Open Database License (http://opendatacommons.org/licenses/odbl/1.0/). The Open Database License (ODbL) is a license agreement intended to allow users to freely share, modify, and use this Dataset while maintaining this same freedom for others, provided that the original source and author(s) are credited.Andrés Martínez-Aquino, Jhonny Geovanny García-Teh, Fadia Sara Ceccarelli, Rogelio Aguilar-Aguilar, Víctor Manuel Vidal-Martínez, Ma. Leopoldina Aguirre-Macedo

Supplementary material 2 Figure S1 Explanation note Scanning electron microscopy (SEM) images of *Xystretrum solidum* (from five specimens collected in the Northern Yucatan Peninsula, Mexico). (**A**) Oral sucker, showing 13 pairs of papillae surrounding margin of oral sucker (locality: Chelem). (**B**) Stylet scar, showing 2 pairs of surrounding papillae and one lateral (dark and light green arrowhead, respectively) (locality: Ría Lagartos). (**C**) Forebody, showing 6 pairs of robust papillae (white arrowhead) (locality: Chuburna). (**D**) Oral sucker, showing 13 pairs of papillae surrounding anterolateral margin (locality: Chuburna). (**E**) Oral sucker, showing single pair in upper region inside oral sucker (red arrowhead) (locality: Chuburna). (**F**) Genital pore detail (locality: Chuburna). (**G**) Ventral sucker, showing long papillae on inner margin (white arrowhead) (locality: Chuburna). (**H**) Details of hindbody corrugation and demarcation of folds (locality: Chuburna). (**I**) Details of “pseudopapilles” on smooth lateral margins of hindbody (white arrowhead) (locality: Chuburna). (**J**) Oral sucker, showing 13 pairs of papillae surrounding margin of oral sucker (locality: Celestún). Colors of arrowheads in A, D and J as follows: Yellow arrowhead = 5 pairs of papillae on interior margin, surrounding mouth; Dark blue arrowhead = one pair of papillae posterolateral to interior margin; Light blue arrowhead = three pairs of papillae anterolateral to interior margin; Dark green arrowhead = 2 pairs of papillae on stylet scar; Light green arrowhead = one pair of papillae lateral to stylet scar; Black arrowhead = one pair of papillae on posterior external margin of oral sucker. Scale bars: 100 µm (**A, J**); 50 µm (**B, D, E, F, G**); 200 µm (**C, I**); 20 µm (**H**); 200 µm (**I**). https://binary.pensoft.net/file/398500This dataset is made available under the Open Database License (http://opendatacommons.org/licenses/odbl/1.0/). The Open Database License (ODbL) is a license agreement intended to allow users to freely share, modify, and use this Dataset while maintaining this same freedom for others, provided that the original source and author(s) are credited.Andrés Martínez-Aquino, Jhonny Geovanny García-Teh, Fadia Sara Ceccarelli, Rogelio Aguilar-Aguilar, Víctor Manuel Vidal-Martínez, Ma. Leopoldina Aguirre-Macedo
