# Supplementary figures and images for: New morphological and molecular data for Xystretrum solidum (Gorgoderidae, Gorgoderinae) from Sphoeroides testudineus (Tetraodontiformes, Tetraodontidae) in Mexican waters
Source: Zookeys. 2020 Apr 8;925:141–61. doi: 10.3897/zookeys.925.49503 (PMC7160187; doi:10.3897/zookeys.925.49503)

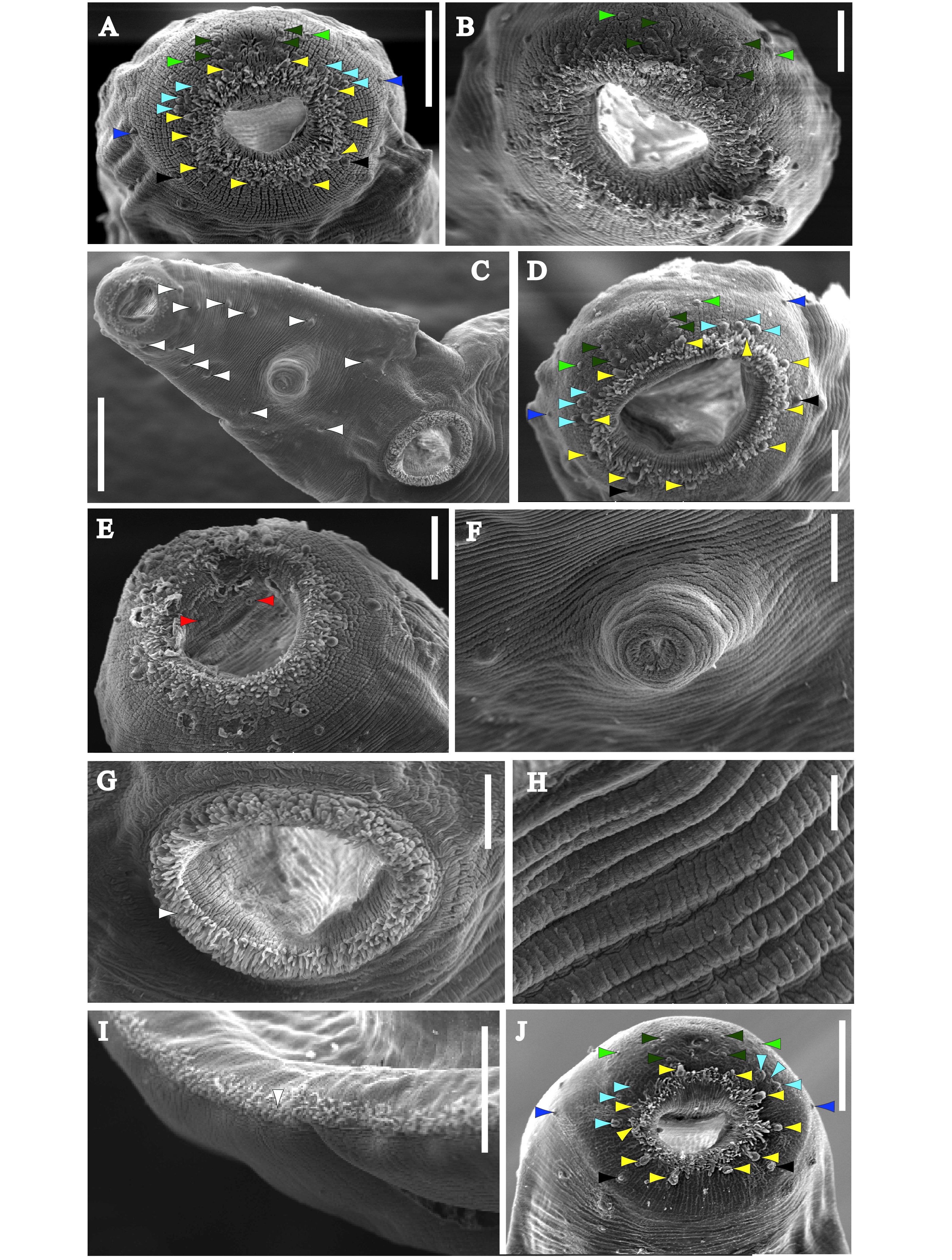

Supplement: Supplementary material 2 — Figure S1 [file zookeys-925-141-s002.jpg]
